# Supplementary figures and images for: Comparative mitogenomic analysis of mirid bugs (Hemiptera: Miridae) and evaluation of potential DNA barcoding markers
Source: PeerJ. 2017 Aug 3;5:e3661. doi: 10.7717/peerj.3661 (PMC5545108; doi:10.7717/peerj.3661)

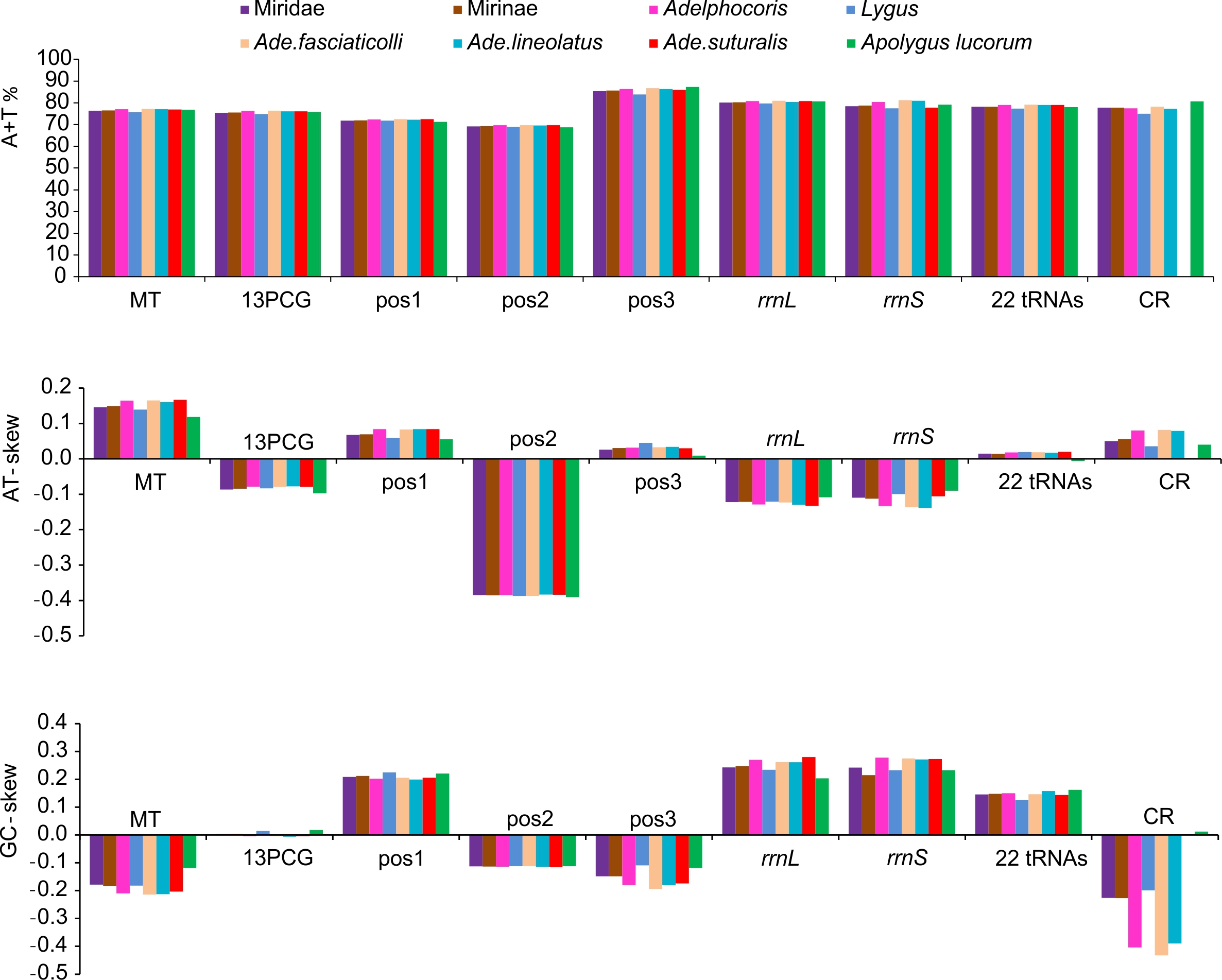

Supplement: Figure S1 [file peerj-05-3661-s001.jpg]

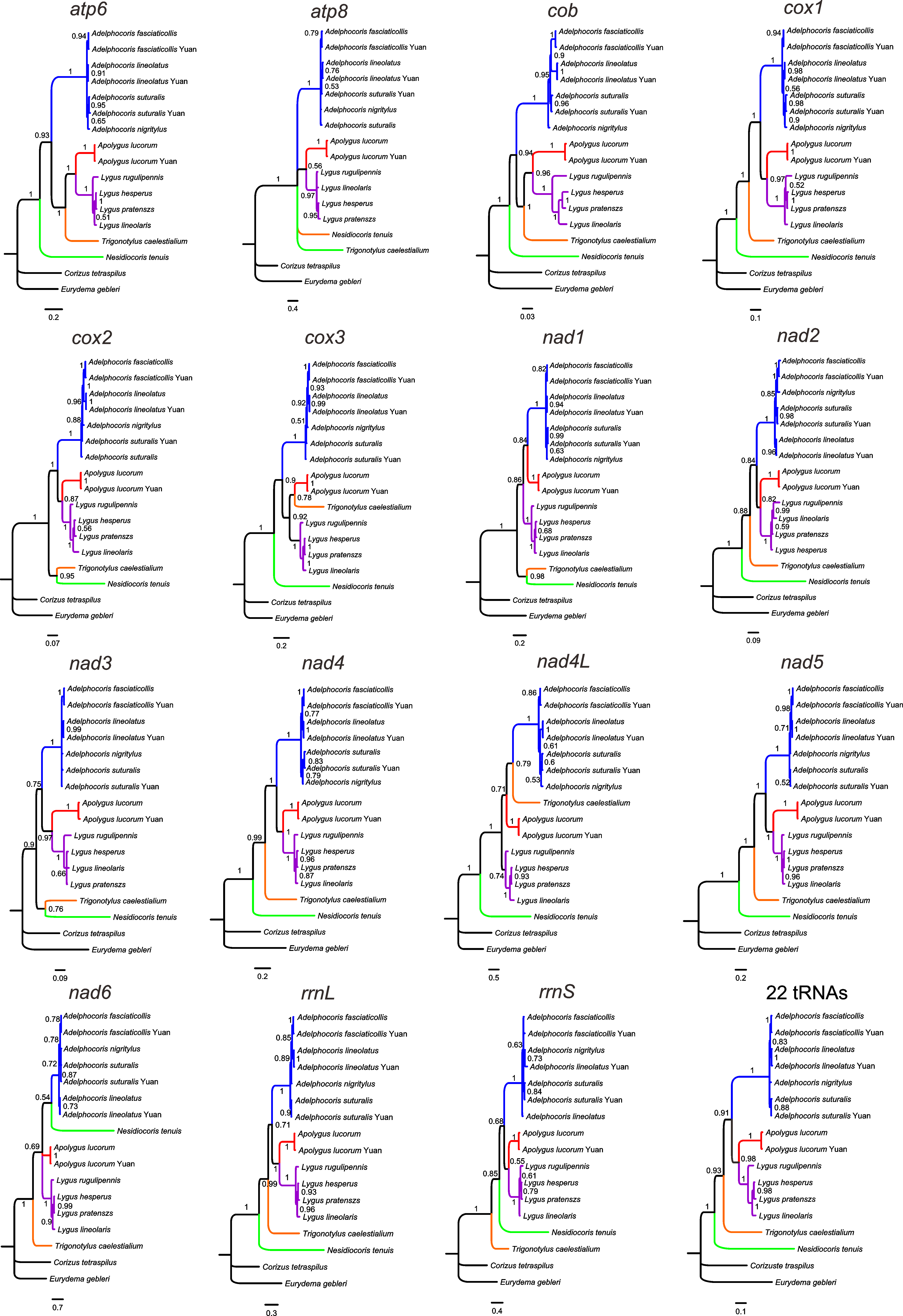

Supplement: Figure S2 — Numbers on branches are Bayesian posterior probabilities. [file peerj-05-3661-s002.jpg]

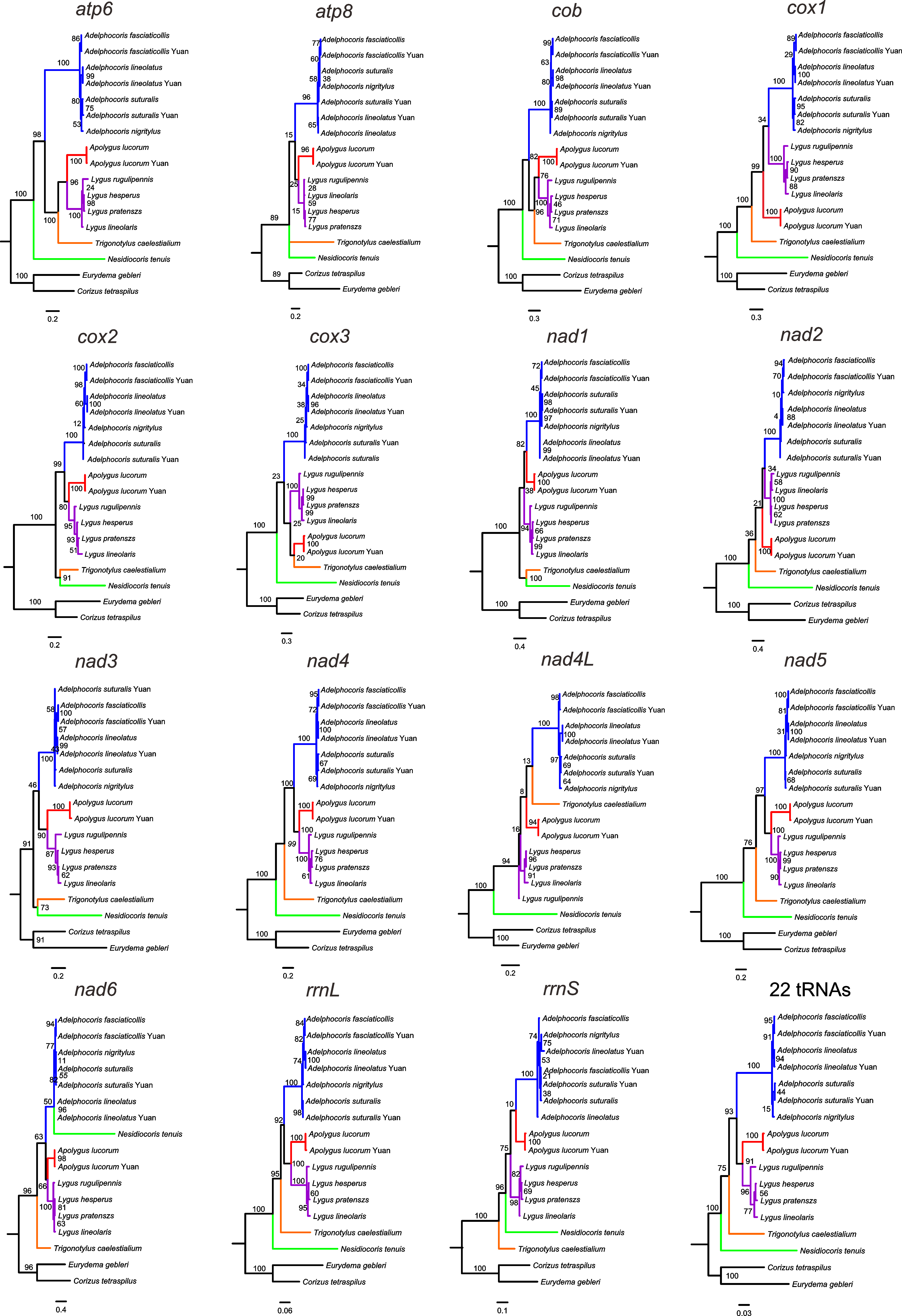

Supplement: Figure S3 — Numbers on branches are bootstrap support values. [file peerj-05-3661-s003.jpg]

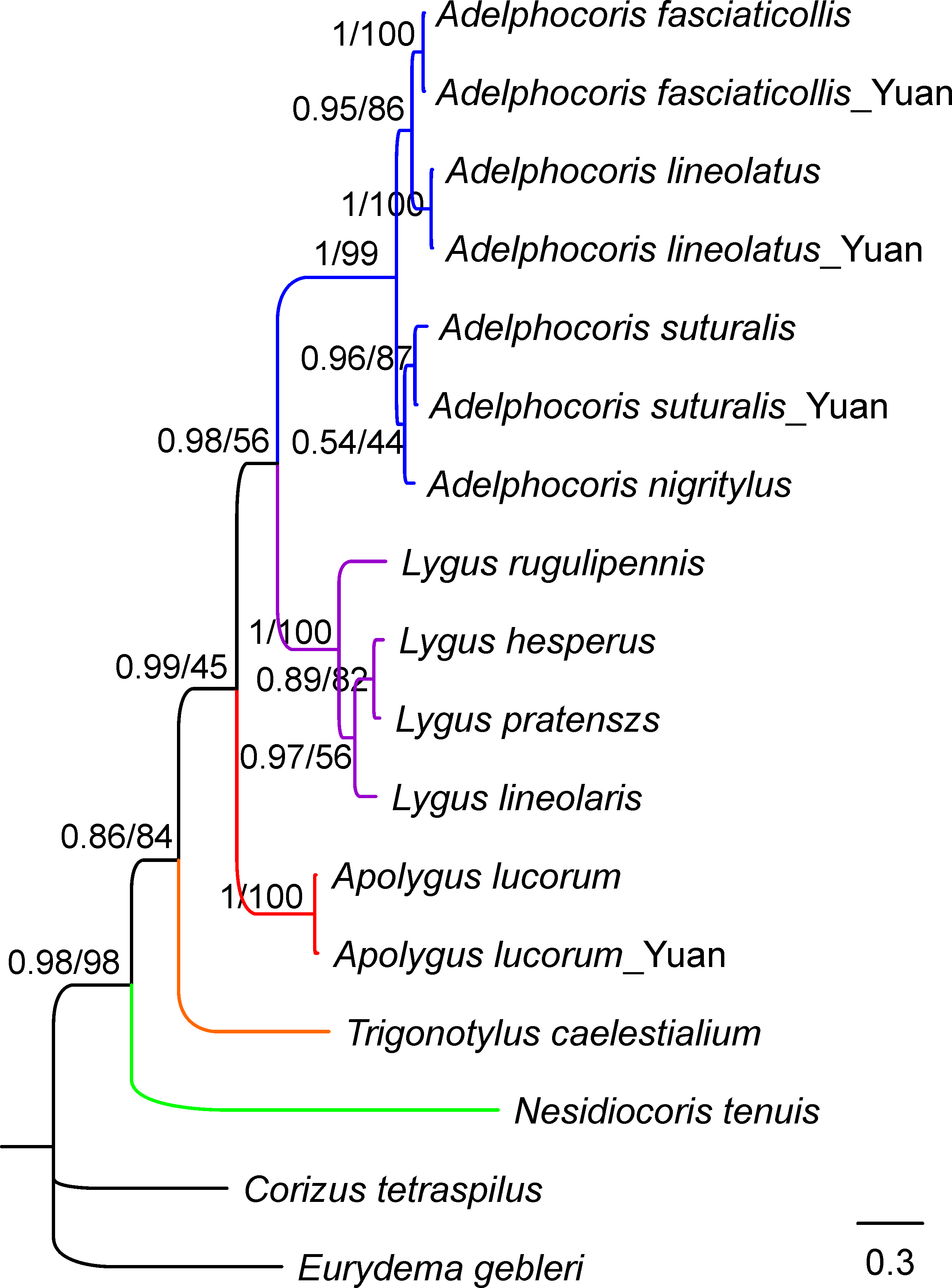

Supplement: Figure S4 — Numbers on branches are Bayesian posterior probabilities (left) and Bootstrap values (right). [file peerj-05-3661-s004.jpg]
