# Supplementary material for: Comparative mitogenomic analysis of mirid bugs (Hemiptera: Miridae) and evaluation of potential DNA barcoding markers
Source: PeerJ. 2017 Aug 3;5:e3661. doi: 10.7717/peerj.3661 (PMC5545108; doi:10.7717/peerj.3661)
Supplement: Table S2 — The best partitioning schemes and substitution models were selected by PartitionFinder for the P123 and P123RT datasets, and each of 13 PCGs. The best substitution models for rrnL, rrnS, 22 tRNAs and cox1-barcode sequences were determined by jModelTest. [file peerj-05-3661-s006.doc]

**Table S2. Evolutionary models for each dataset. The best partitioning schemes and substitution models were selected by PartitionFinder for the P123 and P123RT datasets, and each of 13 PCGs. The best substitution models for *rrnL*, *rrnS*, 22 tRNAs and *cox1*-barcode sequences were determined by jModelTest.**

| **Dataset** | **Subset** | **Subset Partitions** | **Model** |
| --- | --- | --- | --- |
| P123 | P1 | atp6_pos1, atp8_pos2, cob_pos1, cox1_pos1, cox2_pos1, cox3_pos1, nad3_pos1 | GTR+I+G |
|  | P2 | atp6_pos2, nad2_pos2, nad4L_pos2, nad4_pos2, nad5_pos2, nad6_pos2 | GTR+G |
|  | P3 | atp6_pos3, atp8_pos3, cob_pos3, cox1_pos3, cox2_pos3, cox3_pos3, nad2_pos3, nad3_pos3, nad6_pos3 | TrN+I+G |
|  | P4 | atp8_pos1, nad1_pos1, nad2_pos1, nad4L_pos1, nad4_pos1, nad5_pos1, nad6_pos1 | GTR+I+G |
|  | P5 | cob_pos2, cox1_pos2, cox2_pos2, cox3_pos2, nad1_pos2, nad3_pos2 | TVM+I+G |
|  | P6 | nad1_pos3, nad4L_pos3, nad4_pos3, nad5_pos3 | HKY+I+G |
| P123RT | P1 | 22tRNA, atp6_pos1, atp8_pos2, cob_pos1, cox1_pos1, cox2_pos1, cox3_pos1, nad1_pos1, nad3_pos1 | GTR+I+G |
|  | P2 | atp6_pos2, nad2_pos2, nad4L_pos2, nad4_pos2, nad5_pos2, nad6_pos2 | GTR+G |
|  | P3 | atp6_pos3, atp8_pos3, cob_pos3, cox1_pos3, cox2_pos3, cox3_pos3, nad2_pos3, nad3_pos3, nad6_pos3 | TrN+I+G |
|  | P4 | atp8_pos1, nad2_pos1, nad4L_pos1, nad4_pos1, nad5_pos1, nad6_pos1, rrnL, rrnS | GTR+I+G |
|  | P5 | cob_pos2, cox1_pos2, cox2_pos2, cox3_pos2, nad1_pos2, nad3_pos2 | TVM+I+G |
|  | P6 | nad1_pos3, nad4L_pos3, nad4_pos3, nad5_pos3 | HKY+I+G |
| *atp6* | P1 | atp6_pos1, atp6_pos2 | K81uf+G |
|  | P2 | atp6_pos3 | HKY+I+G |
| *atp8* | P1 | atp8_pos1, atp8_pos2, atp8_pos3 | HKY+G |
| *cob* | P1 | cob_pos1, cob_pos2 | TIM+G |
|  | P2 | cob_pos3 | TrN+I+G |
| *cox1* | P1 | cox1_pos1, cox1_pos2 | TIM+G |
|  | P2 | cox1_pos3 | HKY+G |
| *cox2* | P1 | cox2_pos1, cox2_pos2 | K81uf+G |
|  | P2 | cox2_pos3 | TrN+G |
| *cox3* | P1 | cox3_pos1, cox3_pos2 | K81uf+G |
|  | P2 | cox3_pos3 | HKY+I+G |
| *nad1* | P1 | nad1_pos1, nad1_pos2 | K81uf+G |
|  | P2 | nad1_pos3 | HKY+G |
| *nad2* | P1 | nad2_pos1, nad2_pos2 | TVM+G |
|  | P2 | nad2_pos3 | K81uf |
| *nad3* | P1 | nad3_pos1, nad3_pos2 | K81uf+G |
|  | P2 | nad3_pos3 | HKY+G |
| *nad4* | P1 | nad4_pos1, nad4_pos2 | K81uf+G |
|  | P2 | nad4_pos3 | TrN+G |
| *nad4L* | P1 | nad4L_pos1, nad4L_pos2, nad4L_pos3 | K81uf+G |
| *nad5* | P1 | nad5_pos1, nad5_pos2 | K81uf+G |
|  | P2 | nad5_pos3 | HKY+G |
| *nad6* | P1 | nad6_pos1, nad6_pos2, nad6_pos3 | HKY+G |
| *rrnL* | P1 | *rrnL* | TVM+G |
| *rrnS* | P1 | *rrnS* | TVM+I+G |
| 22 tRNAs | P1 | 22 tRNAs | TVM+G |
| *cox1*-barcode | P1 | *cox1*-barcode | TIM2+I+G |
