# Supplementary material for: Comparative mitogenomic analysis of mirid bugs (Hemiptera: Miridae) and evaluation of potential DNA barcoding markers
Source: PeerJ. 2017 Aug 3;5:e3661. doi: 10.7717/peerj.3661 (PMC5545108; doi:10.7717/peerj.3661)
Supplement: Table S5 [file peerj-05-3661-s009.doc]

**Table S5.** Length variation (bp) of 13 protein-coding genes, *rrnL* and *rrnS* among the 15 mirid mitogenomes.

| Species | *atp6* | *atp8* | *cob* | *cox1* | *cox2* | *cox3* | *nad1* | *nad2* | *nad3* | *nad4* | *nad4L* | *nad5* | *nad6* | *rrnL* | *rrnS* |
| --- | --- | --- | --- | --- | --- | --- | --- | --- | --- | --- | --- | --- | --- | --- | --- |
| *Adelphocoris fasciaticollis* | 669 | 156 | 1131 | 1533 | 678 | 783 | 924 | 993 | 351 | 1326 | 303 | 1695 | 486 | 1229 | 793 |
| *Adelphocoris fasciaticollis_*Yuan | 669 | 156 | 1131 | 1533 | 678 | 783 | 924 | 993 | 351 | 1326 | 303 | 1695 | 486 | 1229 | – |
| *Adelphocoris lineolatus* | 669 | 156 | 1131 | 1533 | 678 | 783 | 924 | 993 | 351 | 1326 | 303 | 1695 | 486 | 1231 | 789 |
| *Adelphocoris lineolatus_*Yuan | 669 | 156 | 1131 | 1533 | 678 | 783 | 924 | 993 | 351 | 1326 | 303 | 1695 | 486 | 1230 | 797 |
| *Adelphocoris nigritylus* | 669 | 156 | 1131 | 1533 | 678 | 783 | 924 | 993 | 351 | 1326 | 303 | 1695 | 486 | 1231 | 789 |
| *Adelphocoris suturalis* | 669 | 156 | 1131 | 1533 | 678 | 783 | 924 | 993 | 351 | 1326 | 303 | 1695 | 486 | 1230 | – |
| *Adelphocoris suturalis_*Yuan | 669 | 156 | 1131 | 1533 | 678 | 783 | 924 | 993 | 351 | 1326 | 303 | 1695 | 486 | 1229 | – |
| *Apolygus lucorum* | 669 | 156 | 1131 | 1533 | 678 | 783 | 924 | 1005 | 351 | 1326 | 303 | 1707 | 483 | 1247 | 794 |
| *Apolygus lucorum_*Yuan | 669 | 156 | 1131 | 1533 | 678 | 783 | 924 | 1005 | 351 | 1326 | 303 | 1707 | 483 | 1248 | 794 |
| *Lygus hesperus* | 669 | 159 | 1131 | 1533 | 678 | 783 | 924 | 1005 | 351 | 1326 | 303 | 1698 | 486 | 1248 | 844 |
| *Lygus lineolaris* | 669 | 159 | 1128 | 1533 | 678 | 783 | 924 | 1005 | 351 | 1326 | 303 | 1698 | 486 | 1248 | 844 |
| *Lygus pratenszs* | 669 | 159 | 1131 | 1533 | 678 | 783 | 924 | 1005 | 351 | 1326 | 303 | 1698 | 486 | 1249 | – |
| *Lygus rugulipennis* | 669 | 159 | 1131 | 1533 | 678 | 783 | 924 | 1005 | 351 | 1326 | 303 | 1698 | 486 | 1250 | 824 |
| *Nesidiocoris tenuis* | 672 | 156 | 1131 | 1533 | 675 | 783 | 924 | 912 | 351 | 1323 | 288 | 1680 | 489 | 1221 | 798 |
| *Trigonotylus caelestialium* | 669 | 156 | 1131 | 1533 | 678 | 783 | 924 | 1005 | 351 | 1326 | 306 | 1701 | 483 | 1238 | – |
| Mean | 669.2 | 156.8 | 1130.8 | 1533 | 677.8 | 783 | 924 | 993.2 | 351 | 1325.8 | 302.2 | 1696.8 | 485.6 | 1237.2 | 806.6 |
| SD | 0.77 | 1.37 | 0.77 | 0.00 | 0.77 | 0.00 | 0.00 | 23.25 | 0.00 | 0.77 | 4.00 | 6.19 | 1.55 | 9.98 | 22.08 |
