# Supplementary material for: Comparative mitogenomic analysis of mirid bugs (Hemiptera: Miridae) and evaluation of potential DNA barcoding markers
Source: PeerJ. 2017 Aug 3;5:e3661. doi: 10.7717/peerj.3661 (PMC5545108; doi:10.7717/peerj.3661)
Supplement: Table S6 [file peerj-05-3661-s010.doc]

**Table S6.** Evolutionary analyses of *cox1*-barcode sequences from the 15 mirid mitochondrial genomes.

|  | *cox1* barcode | | | |
| --- | --- | --- | --- | --- |
|  | K2P | Ka | Ks | Ka/Ks |
| Miridae | 0.1245 | 0.1569 | 0.0166 | 9.45 |
| Mirinae | 0.1063 | 0.1334 | 0.0115 | 11.60 |
| Mirini | 0.0951 | 0.1216 | 0.0080 | 15.20 |
| *Adelphocoris* | 0.0216 | 0.0274 | 0.0021 | 13.05 |
| *Lygus* | 0.0420 | 0.0513 | 0.0075 | 6.84 |
| *Adelphocoris fasciaticollis* | 0.0000 | 0.0000 | 0.0000 | – |
| *Adelphocoris lineolatus* | 0.0000 | 0.0000 | 0.0000 | – |
| *Adelphocoris suturalis* | 0.0092 | 0.0099 | 0.0074 | 1.34 |
| *Apolygus lucorum* | 0.0015 | 0.0020 | 0.0000 | – |
