# Supplementary material for: Comparative mitogenomic analysis of mirid bugs (Hemiptera: Miridae) and evaluation of potential DNA barcoding markers
Source: PeerJ. 2017 Aug 3;5:e3661. doi: 10.7717/peerj.3661 (PMC5545108; doi:10.7717/peerj.3661)
Supplement: Table S7 [file peerj-05-3661-s011.doc]

**Table S7.** Evolutionary analyses of *cox1*-barcode sequences of Miridae available on the GenBank.

| **genus** | **Haplotype** | **Ka** | **Ks** | **Ka/Ks** |
| --- | --- | --- | --- | --- |
| *Acetropis* | 1 | **—** | **—** | **—** |
| *Adelphocoris* | 21 | 0.042 | 0.001 | 32.54 |
| *Agnocoris* | 13 | 0.064 | 0.001 | 64 |
| *Alloeotomus* | 1 | **—** | **—** | **—** |
| *Amblytylus* | 1 | **—** | **—** | **—** |
| *Aoplonema* | 3 | 0.032 | 0 | **—** |
| *Apolygus* | 10 | 0.025 | 0 | **—** |
| *Atomoscelis* | 5 | 0.002 | 0 | **—** |
| *Atractotomus* | 8 | 0.186 | 0.01 | 18.6 |
| *Blepharidopterus* | 12 | 0.113 | 0.021 | 5.38 |
| *Bothynotus* | 1 | **—** | **—** | **—** |
| *Brooksetta* | 1 | **—** | **—** | **—** |
| *Bryocoris* | 3 | 0 | 0 | **—** |
| *Calocoris* | 2 | 0.262 | 0 | **—** |
| *Campylomma* | 3 | 0 | 0 | **—** |
| *Campyloneura* | 1 | **—** | **—** | **—** |
| *Capsodes* | 2 | 0.002 | 0 | **—** |
| *Capsus* | 1 | **—** | **—** | **—** |
| *Ceratocapsus* | 7 | 0.142 | 0.012 | 11.83 |
| *Charagochilus* | 8 | 0.091 | 0.022 | 4.14 |
| *Chlamydatus* | 19 | 0.167 | 0.016 | 10.44 |
| *Chlamyopsallus* | 1 | **—** | **—** | **—** |
| *Closterotomus* | 11 | 0.126 | 0.009 | 14 |
| *Coccobaphes* | 1 | **—** | **—** | **—** |
| *Collaria* | 5 | 0.003 | 0 | **—** |
| *Compsidolon* | 4 | 0.121 | 0.008 | 15.13 |
| *Coquillettia* | 2 | 0.004 | 0 | **—** |
| *Corticoris* | 2 | 0.029 | 0.008 | 3.63 |
| *Cremnocephalus* | 2 | 0.182 | 0.024 | 7.58 |
| *Creontiades* | 2 | 0.16 | 0.008 | 20 |
| *Criocoris* | 5 | 0.121 | 0.018 | 6.72 |
| *Cyrtorhinus* | 3 | 0.004 | 0 | **—** |
| *Deraeocoris* | 40 | 0.197 | 0.019 | 10.37 |
| *Dichaetocoris* | 2 | 0.002 | 0 | **—** |
| *Dichrooscytus* | 8 | 0.142 | 0.014 | 10.14 |
| *Dicyphus* | 16 | 0.217 | 0.01 | 21.7 |
| *Eminoculus* | 1 | **—** | **—** | **—** |
| *Europiella* | 8 | 0.13 | 0.004 | 32.5 |
| *Eurycolpus* | 3 | 0 | 0 | **—** |
| *Fulvius* | 1 | **—** | **—** | **—** |
| *Globiceps* | 5 | 0.011 | 0.005 | 2.2 |
| *Hadrodemus* | 3 | 0.027 | 0 | **—** |
| *Hadronema* | 3 | 0.016 | 0 | **—** |
| *Halticus* | 6 | 0.183 | 0.026 | 7.04 |
| *Harpocera* | 1 | **—** | **—** | **—** |
| *Helopeltis* | 3 | 0.11 | 0.011 | 10 |
| *Henrylygus* | 2 | 0.004 | 0 | **—** |
| *Horistus* | 1 | **—** | **—** | **—** |
| *Hyaliodes* | 4 | 0 | 0 | **—** |
| *Ilnacora* | 1 | **—** | **—** | **—** |
| *Ilnacorella* | 3 | 0.008 | 0 | **—** |
| *Irbisia* | 17 | 0.143 | 0.01 | 14.3 |
| *Keltonia* | 1 | **—** | **—** | **—** |
| *Knightomiris* | 1 | **—** | **—** | **—** |
| *Labopidea* | 5 | 0.125 | 0.015 | 8.33 |
| *Labops* | 7 | 0.143 | 0.016 | 8.94 |
| *Leptopterna* | 14 | 0.093 | 0.003 | 31 |
| *Liocoris* | 2 | 0 | 0 | **—** |
| *Litomiris* | 5 | 0.081 | 0.003 | 27 |
| *Lopidea* | 14 | 0.107 | 0.005 | 21.4 |
| *Lopus* | 1 | **—** | **—** | **—** |
| *Lygidea* | 1 | **—** | **—** | **—** |
| *Lygocoris* | 31 | 0.133 | 0.017 | 7.82 |
| *Lygus* | 126 | 0.02 | 0.001 | 20 |
| *Macrolophus* | 5 | 0.254 | 0.041 | 6.2 |
| *Macrotylus* | 4 | 0.198 | 0.012 | 16.5 |
| *Malacocoris* | 2 | 0.002 | 0 | **—** |
| *Mecomma* | 12 | 0.125 | 0.021 | 5.95 |
| *Megaloceroea* | 3 | 0.018 | 0 | **—** |
| *Megalocoleus* | 1 | **—** | **—** | **—** |
| *Melanotrichus* | 8 | 0.17 | 0.016 | 10.63 |
| *Melymacra* | 1 | **—** | **—** | **—** |
| *Metriorrhynchomiris* | 2 | 0.015 | 0 | **—** |
| *Microtechnites* | 1 | **—** | **—** | **—** |
| *Mimoceps* | 2 | 0.006 | 0 | **—** |
| *Miridae sp.* | 23 | 0.255 | 0.031 | 8.23 |
| *Mirinae sp.* | 1 | **—** | **—** | **—** |
| *Miris* | 2 | 0.002 | 0 | **—** |
| *Monalocoris* | 12 | 0.046 | 0 | **—** |
| *Monosynamma* | 5 | 0.036 | 0.009 | 4 |
| *Myrmecoris* | 1 | **—** | **—** | **—** |
| *Neolygus* | 9 | 0.072 | 0 | **—** |
| *Nesidiocoris* | 8 | 0.063 | 0.014 | 4.5 |
| *Neurocolpus* | 5 | 0.073 | 0.003 | 24.33 |
| *Noctuocoris* | 1 | **—** | **—** | **—** |
| *Notostira* | 5 | 0.029 | 0 | **—** |
| *Oligotylus* | 3 | 0.016 | 0 | **—** |
| *Oncotylus* | 2 | 0 | 0.008 | 0 |
| *Orectoderus* | 5 | 0.028 | 0.003 | 9.33 |
| *Orthocephalus* | 5 | 0.161 | 0.022 | 7.32 |
| *Orthonotus* | 3 | 0.04 | 0.015 | 2.67 |
| *Orthops* | 10 | 0.06 | 0 | **—** |
| *Orthotylus* | 36 | 0.178 | 0.014 | 12.71 |
| *Pachytomella* | 1 | **—** | **—** | **—** |
| *Pamillia* | 1 | **—** | **—** | **—** |
| *Pantilius* | 3 | 0 | 0 | **—** |
| *Pappus* | 2 | 0.024 | 0.008 | 3 |
| *Paraproba* | 6 | 0.141 | 0.02 | 7.05 |
| *Parapsallus* | 1 | **—** | **—** | **—** |
| *Parthenicus* | 6 | 0.216 | 0.022 | 9.82 |
| *Phoenicocoris* | 15 | 0.179 | 0.017 | 10.53 |
| *Phylinae* | 2 | 0.263 | 0.001 | 263 |
| *Phylus* | 3 | 0.097 | 0 | **—** |
| *Phytocoris* | 81 | 0.145 | 0.014 | 10.36 |
| *Piceophylus* | 3 | 0.009 | 0.005 | 1.8 |
| *Pilophorus* | 39 | 0.149 | 0.015 | 9.93 |
| *Pinalitus* | 6 | 0.14 | 0.008 | 17.5 |
| *Pinophylus* | 1 | **—** | **—** | **—** |
| *Pithanus* | 4 | 0.006 | 0.004 | 1.5 |
| *Plagiognathus* | 45 | 0.12 | 0.006 | 20 |
| *Platylygus* | 1 | **—** | **—** | **—** |
| *Plesiodema* | 3 | 0.048 | 0 | **—** |
| *Poecilocapsus* | 4 | 0.034 | 0.004 | 8.5 |
| *Polymerus* | 17 | 0.156 | 0.016 | 9.75 |
| *Prepops* | 6 | 0.054 | 0 | **—** |
| *Proba* | 1 | **—** | **—** | **—** |
| *Psallovius* | 9 | 0.068 | 0.008 | 8.5 |
| *Psallus* | 19 | 0.167 | 0.012 | 13.92 |
| *Pseudatomoscelis* | 5 | 0.009 | 0 | **—** |
| *Pseudoloxops* | 1 | **—** | **—** | **—** |
| *Pygovepres* | 1 | **—** | **—** | **—** |
| *Reuteria* | 1 | **—** | **—** | **—** |
| *Reuteroscopus* | 3 | 0.036 | 0 | **—** |
| *Rhabdomiris* | 4 | 0.005 | 0 | **—** |
| *Rhinacloa* | 2 | 0.002 | 0 | **—** |
| *Rhinocapsus* | 2 | 0.002 | 0 | **—** |
| *Salicarus* | 1 | **—** | **—** | **—** |
| *Salicopsallus* | 1 | **—** | **—** | **—** |
| *Salignus* | 2 | 0.008 | 0 | **—** |
| *Schaffneria* | 1 | **—** | **—** | **—** |
| *Slaterocoris* | 11 | 0.111 | 0.007 | 15.86 |
| *Stenodema* | 26 | 0.124 | 0.015 | 8.27 |
| *Stenotus* | 5 | 0.008 | 0 | **—** |
| *Strongylocoris* | 1 | **—** | **—** | **—** |
| *Systellonotus* | 1 | **—** | **—** | **—** |
| *Taedia* | 4 | 0.008 | 0 | **—** |
| *Taylorilygus* | 1 | **—** | **—** | **—** |
| *Teleorhinus* | 2 | 0.004 | 0 | **—** |
| *Teratocoris* | 4 | 0.075 | 0 | **—** |
| *Trigonotylus* | 22 | 0.094 | 0.013 | 7.23 |
| *Tropidosteptes* | 5 | 0.113 | 0.021 | 5.38 |
| *Tupiocoris* | 14 | 0.1 | 0.005 | 20 |
| *Tuxedo* | 2 | 0.204 | 0 | **—** |
| *Tytthus* | 1 | **—** | **—** | **—** |
| All_Miridae | 1076 | 0.213 | 0.024 | 8.88 |
